# Acidifying bacteria inhibit pathogenic bacteria more strongly with increasing glucose

**DOI:** 10.1093/ismejo/wrag170

**Published:** 2026-06-27

**Authors:** Andrea R Dos Santos, Clément Vulin, Sara Mitri

**Affiliations:** Department of Fundamental Microbiology, University of Lausanne, Quartier Unil-Sorge, Bâtiment Biophore, CH-1015 Lausanne, Switzerland; Department of Fundamental Microbiology, University of Lausanne, Quartier Unil-Sorge, Bâtiment Biophore, CH-1015 Lausanne, Switzerland; Department of Fundamental Microbiology, University of Lausanne, Quartier Unil-Sorge, Bâtiment Biophore, CH-1015 Lausanne, Switzerland

**Keywords:** control, pH, antibiotic, pathogens

## Abstract

Classical microbiology has focused on directly suppressing pathogens using drugs, ignoring other harmless microbial species living alongside them. We now have a much better understanding of how species interact and affect one another’s growth within microbial communities, e.g. through chemical production. Here we capitalize on this understanding to demonstrate how one can manipulate and control the strength of interactions between bacterial species, and combine this with antibiotics to eliminate pathogens. Using experiments and a mathematical model, we first show how *Citrobacter freundii* can suppress the pathogen *Pseudomonas aeruginosa* by reducing the pH, alongside the use of ampicillin. This negative interaction from *C. freundii* to *P. aeruginosa* can be strengthened by increasing glucose concentrations. Our proof-of-concept approach also worked against other pathogens: *Klebsiella pneumoniae* and *Agrobacterium tumefaciens*, and a different competitor species: *Lactobacillus plantarum*, a common probiotic. Overall, we show that taking advantage of the community and chemical context in which microbes live can help to develop efficient strategies to control them. In the medical or agricultural context, this approach can help to eliminate pathogens thereby reducing our reliance on antibiotics.

## Introduction

Pathogens often share their environment inside the host with nonpathogenic microbes. These different species can interact, influencing each other’s growth and survival positively or negatively depending on the species and the context. And yet, traditionally, pathogens have been studied in isolation, resulting in the discovery of many antimicrobial drugs that have become indispensable in treating microbial infections, and have saved millions of lives. But as we approach the crisis of increasing antimicrobial resistance globally [1], there is an urgent need to develop alternative, complementary strategies to effectively clear pathogens.

Thinking about antimicrobial administration in the presence of other microbes has a few important implications: antibiotics may cause collateral damage, killing or inhibiting untargeted competitors of the pathogen and making it easier for the pathogen to rebound [2–5]. Nonpathogenic microbes may instead degrade the antimicrobials, thereby “cross-protecting” the pathogen [6], or enhance their effect to increase pathogen susceptibility to antimicrobials [7]. The presence of other microbes can even impact resistance evolution, as shown in a simple cross-feeding community of *Escherichia coli* and *Salmonella enterica* [8].

Interactions between pathogens and other microbial species need not just be an obstacle for eliminating pathogens or mitigating antimicrobial resistance evolution, though. Can one instead harness our understanding of these interspecies microbial interactions to develop new treatments with improved outcomes? In other words, can we control microbial community members to better compete with pathogens?

Competition is an important component of pathogen resistance. For example, a diverse gut microbiome or certain members of the vaginal microbiome can prevent pathogen colonization [9]. In this latter example, species like *Lactobacilli* acidify the environment, reducing its pH to levels that are lower than the pathogen’s preference, thereby preventing it from invading [10–12]. Such pH-mediated interactions are expected to be common, because all microbes are likely to modify their environmental pH and to respond to such modifications [13]. Because each species’ metabolism and molecular machinery are adapted to function best within a specific pH range, pH modifications can potentially result in dramatic effects on fitness [14], by e.g. modifying the conformation of transport proteins in the bacterial membrane [15, 16]. Because of this generality, environmental acidification (pH reduction) has parallels in other ecosystems, famously in food preservation, where fermentation by lactic acid bacteria leads to strong acidification [17].

Here we connect these ideas to test whether controlling the strength of negative interactions toward a pathogen through acidification can be used to complement the use of antibiotics. In previous work, we have shown that the sign and strength of interactions between two bacterial species can be controlled by modifying the concentration of a single chemical compound in the growth medium [18]. Others have shown that resource concentration can modulate pH changes, in turn affecting species composition [14, 19]. In this study, we apply this same general approach to control the pH changes induced by one species to manipulate a pathogen and increase its susceptibility to antibiotic treatment. Using *Citrobacter freundii* as the acidifying competitor and *Pseudomonas aeruginosa* strain PA14 as the pathogen, we show that *C. freundii* can strongly inhibit *P. aeruginosa* together with the antibiotic ampicillin, and that this effect increases as we provide increasing concentrations of glucose to the co-culture, ultimately replacing the need for ampicillin altogether. A mathematical model allows us to identify key parameters to optimize *P. aeruginosa* inhibition and to predict competitor–pathogen species pairs for which this approach should work. We then experimentally test some of these pairs.

Our work links ideas on colonization resistance, food microbiology, and interspecies interactions to demonstrate an effective strategy that can reduce the use of antibiotics. By taking advantage of the community context in which a pathogen is living, one can control it through the response of co-inhabiting species to resource concentrations, thereby increasing the strength of competition. We call this approach “feeding the enemy’s enemy.”

## Material and methods

### Bacterial strains

The following bacterial species were used in this study: *Pseudomonas aeruginosa* PA14 (kindly donated by Kevin Foster), *Citrobacter freundii* and *Klebsiella pneumoniae* (kindly donated by Peter Küenzi), *Agrobacterium tumefaciens* (kindly donated by Christopher van der Gast and Ian Thompson), and *Lactobacillus plantarum* (kindly donated by Julian Garneau and Pascale Vonaesch). We used a strain of PA14 that contains an mCherry fluorescent tag. We used two strains of *C. freundii*: the strain used in the experiments together with *P. aeruginosa* and *K. pneumoniae* was selected for increased resistance on ampicillin medium. The original isolate that was more sensitive to ampicillin was used in experiments with *A. tumefaciens*.

### Cell cultures

To start bacterial cultures, a single colony was grown overnight (200 rpm, 28$^{\circ }$C) in 10 ml of tryptic soy broth (TSB) and then sub-cultured for 3 h in the same medium at an initial OD$_{600}$ of 0.05. Unless mentioned otherwise, the OD$_{600}$ was adjusted to 0.1, the culture was washed three times with phosphate buffer saline, and then resuspended in the growth medium (depending on the experiment). This bacterial culture was used to inoculate the different conditions of our experiments by diluting it 100$\times $ (final OD$_{600}$ = 0.001). For co-cultures, a final OD$_{600}$ of 0.001 of each species was combined, such that mono- and co-cultures contained similar population sizes of each species. When *C. freundii* was 10$\times $ more, the starting culture was OD$_{600}$ of 1 (final OD$_{600}$ = 0.01). For *L. plantarum*, cells were pre-cultured in Brain Heart Infusion (BHI) instead of TSB. *L. plantarum* cultures were always set to an initial OD$_{600}$ of 0.001, as was *P. aeruginosa* when co-cultured with *L. plantarum*.

Growth and dilution cycles were performed by diluting bacterial cultures every 72 h 100$\times $ into fresh medium corresponding to the same initial glucose and ampicillin concentrations.

### Media recipes

For all cultures involving species other than *L. plantarum*, we used an M9 minimal medium with glucose. To make 1L of medium, we mixed 100 ml of 10$\times $ M9 (pH adjusted to 7.4 with NaOH), 20 ml of 50$\times $ HMB (see [18] for recipes), glucose (e.g. 40 ml of a 0.75 M stock solution to achieve a final concentration of 30 mM) and completed the volume with water. Unless mentioned otherwise, M9 was prepared according to the standard recipe with 60 g/L Na$_{2}$HPO$_{4}$ and 30 g/L KH$_{2}$PO$_{4}$. To manipulate the buffering capacity, these concentrations were halved (30 g/L Na$_{2}$HPO$_{4}$ and 15 g/L KH$_{2}$PO$_{4}$), or doubled (120 g/L Na$_{2}$HPO$_{4}$ and 60 g/L KH$_{2}$PO$_{4}$) to achieve low and high buffering capacity, respectively. TSB and BHI were purchased from BD Difco.

### Measuring population sizes

We measured population sizes based on colony-forming units (CFUs). We first sampled 20 $\mu $g/ml of bacterial culture and serially diluted it down to $10^{-7}$ (in $V = 200$  $\mu $l). A volume of 5 $\mu $l was plated in the form of a drop on tryptic soy agar (TSA) for each 10-fold dilution. Plates were incubated at 28$^{\circ }$C. For cocultures, TSA allowed to distinguish *P. aeruginosa* and *C. freundii* because (i) the strains have distinct colors (*P. aeruginosa* has an mCherry tag making its colonies appear pink) and (ii) *C. freundii* colonies grow after 12 h, whereas *P. aeruginosa* takes between 24 and 48 h to show countable colonies. *Lactobacillus plantarum* grows slowly on TSA, and its colonies were instead counted on De Man, Rogosa, and Sharpe (MRS) plates. The limit of detection was at $10^{2}$ CFUs/ml.

We also quantified bacterial densities in some cases by measuring optical density (OD) at 600 nm in a micro-plate reader (BioTek Synergy H1 at 28$^{\circ }$C with continuous double-orbital shaking), every 30 min for 72 h.

### Minimal inhibitory concentration and growth parameter estimation

In a 96-well plate, the first well of each line was filled with 200 $\mu $l of an ampicillin stock (Ampicillin Sodium Salt *BioChemica*, code A0839, ampicillin powder was diluted in minimal medium with glucose, either 15 mM or 30 mM depending on the experiment). The rest of the wells were filled with minimal medium with glucose (at equal concentrations of glucose, see below). The ampicillin was serially diluted 2x until the penultimate well (final volume: 100 $\mu $l). The bacterial culture was prepared as described above, but at a final OD$_{600}$ of 0.2 in minimal medium with glucose, which was then diluted 100$\times $ in the same medium to obtain a stock culture. We added 100 $\mu $l of this stock to all wells of the 96-well plate so that all wells contained bacteria at an initial OD$_{600}$ of 0.001 and either 15 mM or 30 mM of glucose (final volume: 200 $\mu $ml). Growth was followed by measuring OD$_{600}$ over time as described above. Lag length and final yield (Fig. S1) were determined manually: final yield was the OD$_{600}$ at 72 h and lag length was the timepoint at which the OD$_{600}$ passed a threshold value of 0.09. The effect of pH and ampicillin on final yield and lag length were tested using a linear model in R (e.g. lm(FY$OD600 ~ FY$Amp + FY$pH + FY$Amp*FY$pH)).

### Minimal inhibitory concentrations over a range of pH values

In a 96-well plate, the first well of each line was filled with 200 $\mu $l of ampicillin stock (800 $\mu $g/ml in water). The rest of the wells were filled with 100 $\mu $l of water. We then proceeded to a serial 2x dilution until the penultimate well (final volume: 100 $\mu $l). The bacterial cultures were prepared as described above, however we prepared six bacterial stock cultures at OD 0.002: one per pH condition tested. We thus prepared minimal media with 15 mM of glucose and various concentrations of HCl to obtain a range of pH levels (see Table 1, media concentrated 2x to account for the 2x dilution in the 96-well plate). Each stock culture was then added to one dilution line of the 96-well plate (100 $\mu $l per well). Growth was followed by measuring OD$_{600}$ over time as described above.

**Table 1:** Concentration of HCl added to minimal medium with glucose to obtain a range of pH levels from neutral to acidic.

**HCl [M]**	**Theoretical pH**
0.07	3.5
0.065	4.8
0.06	5.6
0.05	6.2
0.03	6.8
0	7.7

### Use of bromocresol purple to determine the pH of cell cultures

To follow the pH of bacterial cultures over time without using a pH-meter, we used a pH dye: bromocresol purple. This dye is purple at pH $\geq $ 6.8 and yellow at pH $\leq $ 5.2. The purple form of the dye absorbs at 588 nm and can easily be correlated to the pH value of the medium. With the absorption of the dye being so close to 600 nm (absorption of bacterial cells), we measured the density of bacterial cells at 750 nm. An absorption scan on *P. aeruginosa* and *C. freundii* showed that they absorb similarly at 600 and 750 nm. The net absorbance of bromocresol purple is then calculated by subtracting the OD$_{750}$ from the OD$_{588}$. The pH is then calculated using a standard curve mapping the intensity of the absorption at 588 nm to the pH. To do so, we prepared a range of citrate-Na$_{2}$HPO$_{4}$ buffer solutions (Table 2) with known stable pH levels (from pH 4.8 to 7.2). In a 96-well plate, we mixed 180 $\mu $l of each buffer with 20 $\mu $l of bromocresol purple solution 0.01$\%$ (w/w) (in triplicate). We measured the OD$_{588}$, performed a linear regression and extracted the equation to calculate the pH from the absorbance.

**Table 2:** Citrate-Na$_{2}$HPO$_{4}$ buffers in a range of pH levels from neutral to acidic used to produce the standard curve of bromocresol purple at 588 nm.

**pH**	**0.1 M citrate (ml)**	**0.2 M Na$_{2}$HPO$_{4}$ (ml)**
4.8	50.70	49.30
5.0	48.50	51.50
5.2	46.40	53.60
5.4	44.25	55.75
5.6	42.00	58.00
5.8	39.55	60.45
6.0	36.85	63.15
6.2	33.90	66.10
6.4	30.75	69.25
6.6	27.25	72.75
7.2	13.05	86.95

### Mathematical model

The mathematical model is a consumer-resource model based on ordinary differential equations, describing changes in the abundances of the two species, concentrations of glucose and organic acid, and the pH. It is described in detail in the supplement (note S1). The code is available on github here.

## Results

### Ampicillin, low pH, or the presence of *C. freundii* strongly inhibit *P. aeruginosa*

**Figure 1:**
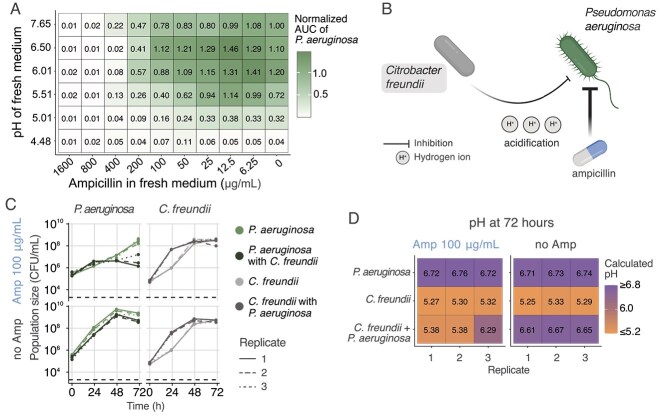
Acidification enhances the effect of ampicillin on the pathogen *Pseudomonas aeruginosa*. (A) MIC assay performed on *P. aeruginosa* in minimal medium with 15 mM glucose, at 10 initial ampicillin concentrations and 6 initial pH levels: from 7.65 (fresh minimal medium, no pH modification) down to 4.48. The fresh medium was acidified using HCl. The OD$_{600}$ was measured every 30 min over 72 h and the AUCs calculated, normalized, and plotted ($n = 3$). (B) *Citrobacter freundii* should inhibit *P. aeruginosa* by acidifying the environment in parallel to inhibition by ampicillin. (C) Growth of *P. aeruginosa* and *C. freundii* in mono- and in co-culture, with or without ampicillin [100 $\mu $g/ml] in minimal medium with 30 mM glucose. Growth was determined by measuring CFU/ml over 72 h ($n = 3$). The dashed line shows the detection limit. (D) pH from all conditions in panel C after 72 h, measured using the absorbance of the pH indicator bromocresol purple at 588 nm (purple above pH = 6.8 and yellow under pH = 5.2, see Methods).

We first sought to confirm previous findings that antibiotics—specifically ampicillin—can more strongly inhibit *P. aeruginosa* at an acidic pH [20–22]. We performed a minimal inhibitory concentration (MIC) assay paired to a pH sensitivity assay where we measured OD$_{600}$ over 72 h for all combinations of 10 initial ampicillin concentrations and 6 initial pH values (Fig. 1A and S1). *Pseudomonas aeruginosa* showed sensitivity to ampicillin (MIC: 200 $\mu $g/ml at neutral pH, Fig. S2B) and to acidification (without ampicillin, growth is at 32$\%$ at pH 5 and no growth is observed below this value, $n = 3$). Gradually reducing the pH reduced the final yield of *P. aeruginosa*, whereas ampicillin lengthened its lag phase (linear model of factors affecting final yield: pH *p*-value < .001; ampicillin *p*-value .22; factors affecting lag phase: pH *p*-value .47, ampicillin *p*-value <.001, see Methods, Fig. S1). Ampicillin and acidification together inhibited *P. aeruginosa*’s overall growth (linear model of factors affecting the area under the growth curve (AUC): ampicillin *p*-value <.001 and pH *p*-value .006, Fig. 1A).

Given that both low pH and ampicillin can negatively affect *P. aeruginosa*, we hypothesized that a species that lowers the pH should inhibit *P. aeruginosa* in the presence of ampicillin (Fig. 1B). To test this, we grew *P. aeruginosa* with *C. freundii*, a common host-associated bacterium. Upon growth alone in a glucose minimal medium, *C. freundii* acidified the medium from a pH of 7.62 down to $\sim $ 5.3 (Fig. 1C and D). Because we needed *C. freundii* to survive the ampicillin treatment long enough to acidify the environment, we isolated a *C. freundii* strain that was more resistant to ampicillin than *P. aeruginosa* (MIC 400 $\mu $g/ml, Fig. S2A, see Methods). To follow the pH of bacterial cultures over time, we used the nontoxic pH dye bromocresol purple whose absorbance at 588 nm can be correlated to the pH of the medium (purple in pH $\geq $ 6.8 and yellow $\leq $ 5.2, see Methods, Fig. S3). To follow the population sizes of both species, we counted their CFUs on TSA, which we could distinguish because *P. aeruginosa* carried an mCherry fluorescent tag.

When grown together without ampicillin, *P. aeruginosa* and *C. freundii* displayed weak interspecies interactions: compared with their growth in mono-culture, *P. aeruginosa* grew slightly worse with *C. freundii* (AUC of mono- versus co-culture, Welch two sample *t*-test, *p*-value .061), whereas *C. freundii* benefited from the presence of *P. aeruginosa* (AUC of mono- versus co-culture, *p*-value .022). Acidification was in a similar range as when *P. aeruginosa* was alone (Fig. 1D, right panel), suggesting that *P. aeruginosa* might consume acidifying by-products produced by *C. freundii*, allowing the pH to remain close to neutral.

We next added ampicillin (100 $\mu $g/ml) to the co-culture, and found that *C. freundii* could acidify the medium and reduce the population size of *P. aeruginosa* by $\sim $100-fold in two out of three replicates (AUC of mono- versus co-culture, Welch two sample *t*-test, *p*-value .0019). In the replicate where *P. aeruginosa* was inhibited the least, the final pH was also the highest (Fig. 1C and D, replicate 3, pH = 6.29), suggesting that *P. aeruginosa* inhibition depends on acidification. In summary, when ampicillin inhibits *P. aeruginosa*, *C. freundii* can lower the pH and further inhibit *P. aeruginosa*.

### Model suggests that larger competitor populations or more glucose can suppress the pathogen

Before running longer-term experiments to explore whether we could eradicate *P. aeruginosa*, we sought to obtain more intuition on how the two-species system would respond to changes in experimental conditions. To this end, we built a consumer-resource model based on the dynamics displayed in Fig. 1, where the pathogen $B_{1}$ (*P. aeruginosa*) and the competitor $B_{2}$ (*C. freundii*) grow on a common resource $C$ (glucose) in presence of an antibiotic (ampicillin). The competitor can produce an organic acid $A$ while consuming $C$, which acidifies the environment, inhibiting the growth of the pathogen. The pathogen consumes glucose as well as some of the produced organic acid and is also inhibited by the antibiotic. The parameters of the model were not fitted to the co-culture data, but were chosen to approximate our experimental observations. The model is described in detail in supplementary note S1.

**Figure 2:**
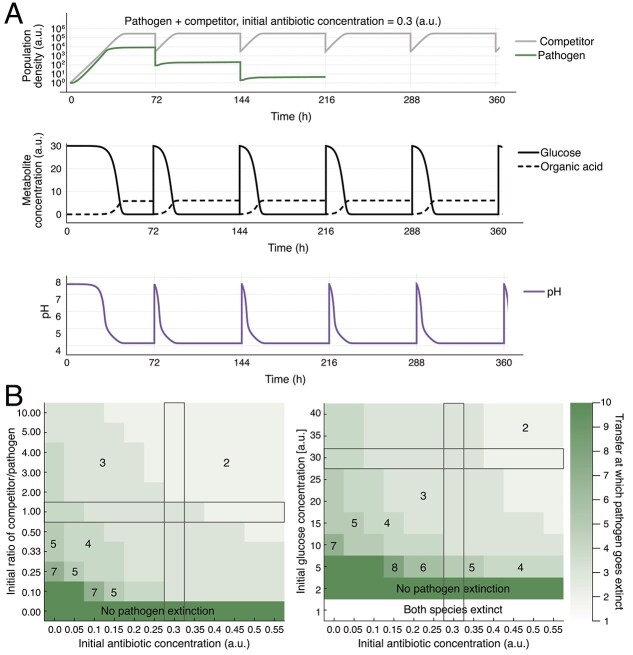
Mathematical model shows how a pathogen goes extinct when grown together with an acidifying competitor. (A) We simulate co-cultures of the pathogen (*P. aeruginosa*) and the competitor (*C. freundii*) in glucose in the presence of antibiotic (ampicillin) for 72 time steps (all arbitrary units, a.u.) followed by a 100$\times $ dilution into fresh glucose and antibiotic (top row, see note S1). Growth and dilution cycles are repeated 10 times (only five are shown). Because the species consume glucose, organic acids accumulate (central row), resulting in a drop in pH (bottom row). (B) We explore whether and how quickly the pathogen can be eliminated under different initial conditions. The pathogen is driven extinct more quickly when (left) the competitor is initially more abundant or (right) when glucose concentrations are higher, and when antibiotic concentrations are higher. The long rectangles show default parameter values (e.g. for simulations in panel A).

Our overall goal is to eliminate *P. aeruginosa* at low ampicillin concentrations to reduce our reliance on antibiotics. To this end, we used the model to identify the conditions under which the pathogen would be excluded if we were to carry out serial transfers or growth-and-dilution cycles (Fig. 2A). We performed two parameter sweeps where we simultaneously varied the antibiotic concentration and either (i) the initial population size of the competitor relative to the pathogen, or (ii) the starting concentration of glucose (Fig. 2B). The model predicts that increasing either competitor population size or glucose concentration can lead to a quicker extinction of the pathogen. If the competitor is absent, the pathogen survives till the end of the simulation under our chosen parameters even with antibiotics. If too little glucose is provided, neither species can grow enough to survive the 100-fold dilution. Overall, however, the model predicts that if the competitor is present, it should not be difficult to eliminate the pathogen in just a few transfers.

### *Citrobacter freundii* eliminates *P. aeruginosa* at high glucose concentration

With our better understanding of the effects of population sizes, glucose, and antibiotic concentrations from the model, we next conducted the transfer experiments. We grew *P. aeruginosa* and *C. freundii* together and in isolation in different concentrations of glucose (15, 26, and 30 mM) and ampicillin (0, 100, 200, 400, and 800 $\mu $g/ml) with $\sim $10$\times $ higher initial OD$_{600}$ of *C. freundii* compared with *P. aeruginosa*. The initial ratio of the two species was chosen after preliminary experiments revealed that it had no significant effect on *P. aeruginosa* survival (generalized linear model, *p*-value .144, Fig. S4 and S5), contrary to the prediction of the model (Fig. 2B, left). Glucose concentrations were chosen such that the pH either remained neutral (15 mM), dropped and recovered back to neutral (26 mM), or dropped and did not recover (30 mM) when *C. freundii* was cultured alone (Fig. S6). Every 3 days for 15 days, we transferred 1$\%$ of the culture into fresh medium with the same initial concentrations of glucose and ampicillin. We mixed the cultures twice per day using a pipette to prevent biofilm formation. CFUs were quantified at the end of each transfer, and pH was followed over time by adding bromocresol purple to the growth medium (see Methods).

**Figure 3:**
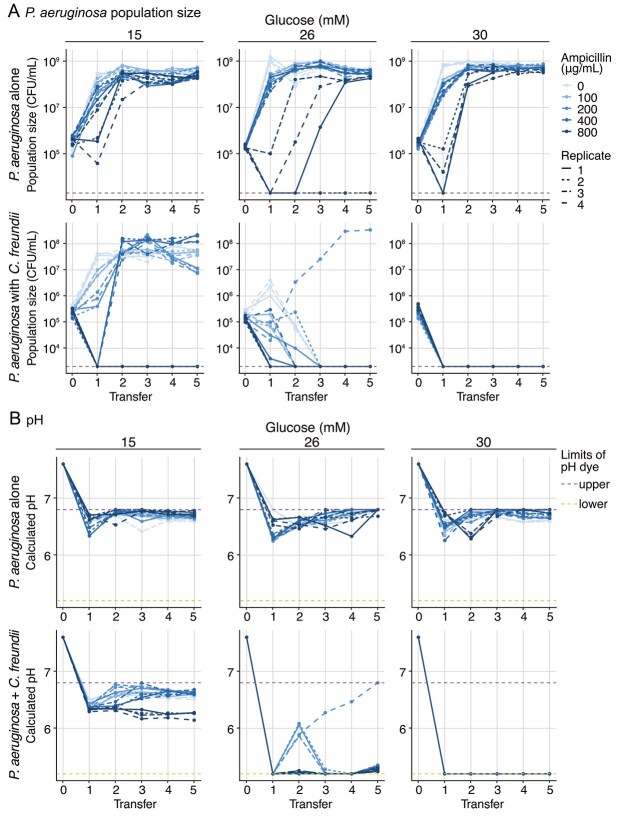
Experiments showing that glucose and ampicillin inhibit *P. aeruginosa* when in co-culture with *C. freundii*. (A) Population size of *P. aeruginosa* in various glucose and ampicillin concentrations when it is alone (top) or co-cultured with *C. freundii* (bottom) over five growth-and-dilution cycles (3 days of growth, 100X dilutions, or 1$\%$ transfers, $n=4$). For the bottom panel, the initial OD$_{600}$ of *C. freundii* (0.01) is 10$\times $ higher than that of *P. aeruginosa* (0.001). Each sub-plot shows the population size of *P. aeruginosa* at the start of the experiment (transfer 0) and before each transfer. The corresponding data for *C. freundii* are shown in Fig. S7. The dashed gray line shows the detection limit. (B) pH calculated based on the absorbance at 588 nm of bromocresol purple (see Methods) at the start of the experiment and before each transfer when *P. aeruginosa* was alone (top) or with *C. freundii* (bottom). The pH of the fresh medium at the initial timepoint was measured with a pH probe ($n = 4$). The dashed purple (top) and yellow (bottom) lines show the upper and lower detection limits of the pH dye, respectively.

When growing alone (Fig. S7), *C. freundii* showed similar growth at all antibiotic concentrations, and acidified more but grew worse at increasing glucose concentrations, suggesting that it also suffered from its own acidification. *P. aeruginosa* instead grew worse at higher ampicillin concentrations, but was able to recover and survive until the end of the experiment in almost every glucose and ampicillin concentration when alone (Fig. 3A, top). When co-cultured with *C. freundii*, however, *P. aeruginosa* dropped below the detection limit in all replicate populations at ampicillin concentrations of 800 $\mu $g/ml and in all replicates at glucose concentrations of 30 mM. *P. aeruginosa* survival was highly dependent on pH, where it was able to grow whenever the pH remained above 6, as long as the ampicillin concentration was low enough (Fig. 3B). Population recovery occurred only once at 26 mM of glucose and not a single time at 30 mM of glucose, where *C. freundii* was able to acidify the environment consistently, even when no ampicillin was added. The role of pH in driving the interaction was further supported by an experiment where we varied the buffering capacity of the minimal medium: decreasing or increasing it strengthened or weakened the negative effect of *C. freundii* on *P. aeruginosa*, respectively (Fig. S8).

In sum, *P. aeruginosa* could be eliminated after the first transfer whenever the initial glucose concentration was high enough, allowing *C. freundii* to cause the pH to drop and remain below 6. When grown alone, the pH remained high such that *P. aeruginosa* survived at all glucose concentrations, and could only be eliminated at high enough concentrations of ampicillin.

### *P. aeruginosa* could survive antibiotic treatment when cultures were not mixed

In the above experiments, we mixed cultures twice per day using a pipette. This choice was informed by earlier experiments, in which cultures were only pipetted when transferred every 3 days. In these first experiments, *C. freundii* consistently drove *P. aeruginosa* to extinction at high glucose concentrations due to a drop in pH, but the antibiotics did not have the expected effect (Fig. S9). Indeed, some *P. aeruginosa* populations survived best at the highest concentration of antibiotics (Fig. S4 and S5) even if their resistance to ampicillin was not greater than the WT (Fig. S10). Visual examination of the wells led us to hypothesize that *P. aeruginosa* was forming biofilms protecting it from the antibiotic, as has been shown in previous work in response to antibiotics [23–25] as well as acidic conditions in cystic fibrosis lungs [26]. To test whether biofilm formation might be protecting *P. aeruginosa*, in a shorter experiment, we mixed the cultures at regular time intervals to destroy any biofilms (Fig. S11), and observed that ampicillin was inhibiting *P. aeruginosa* as we had expected (Fig. S12 and S13). Although this experiment does not prove that biofilms were the culprit, we report on it to encourage future applications of our approach to investigate this issue further.

### Model suggests that this approach can be applied to other species pairs

Our experiments showed that acidification by *C. freundii* at high glucose concentrations could effectively eliminate *P. aeruginosa*. We next wondered whether this could work for other species pairs. To evaluate the generality of our approach, we first used the mathematical model to guide our expectations. This time, in addition to varying the initial antibiotic concentrations, we explored how different properties of the species would change the outcome. For example, in the original model we parameterized the pathogen to grow slightly faster than the competitor (0.45 versus 0.4) to approximate our experimental data. The competitor was also more resistant to the antibiotic than the pathogen and had a higher tolerance for acidity. Would our approach still work if our species pairs did not meet these criteria? And which criteria are needed for success?

**Figure 4:**
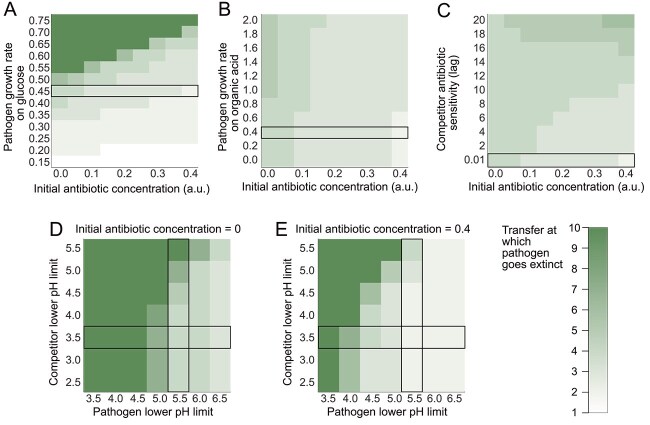
Model suggests the range of species parameters across which we expect this approach to work. Predictions generated by the mathematical model varying different properties of the competitor and pathogen. The black rectangles in each plot show the default parameters used in the simulations shown in Fig. 2. The shade shows the transfer at which the pathogen goes extinct, with 10 meaning it does not until the end of the simulations. (A) If the pathogen grows much faster than the competitor (0.4 in these simulations) on the main supplied carbon source (glucose), more antibiotic will be needed to eliminate it. (B) Its growth rate on the resulting organic acid does not affect the results much. (C) It helps if the competitor is more resistant to the antibiotic, but even if it is not, the pathogen can be eliminated. If too much antibiotic is added and the competitor is sensitive, the approach works less well. (D) The pathogen needs to be less tolerant to the pH compared with the competitor in the absence of antibiotics. (E) Adding antibiotics (concentration 0.4) can compensate for having similar tolerance levels.

The model suggests that the most important properties of the pathogen and competitor species are that (i) the pathogen should not grow much faster than the competitor on the provided carbon source (Fig. 4A) and (ii) the competitor needs to have a higher tolerance to acidity compared with the pathogen (Fig. 4D and E). If either of these criteria are not met (i.e. if the pathogen grows a lot faster than the competitor or the competitor is not much more tolerant to low pH), more antibiotic will be needed to eliminate the pathogen. Whether or not the pathogen consumes the organic acid and the resistance of the competitor to the antibiotic were less important according to our model (Fig. 4B and C). Even though the model is not parameterized using experimental data and is therefore not predictive, finding that the pathogen can be suppressed across a broad parameter range encouraged us to test additional species pairs experimentally. In particular, it suggests that one can omit the antibiotic entirely and use a sensitive competitor. Indeed, antibiotics should even be avoided in these cases, because they can decrease the efficacy of the approach by inhibiting the competitor rather than the pathogen (top right corner of Fig. 4C).

### Testing other species pairs experimentally

The model suggests that the approach should be broadly applicable. To explore this experimentally, we exchanged *P. aeruginosa* for two different pathogens, *Agrobacterium tumefaciens*, a plant pathogen, and *Klebsiella pneumoniae*, another opportunistic human pathogen. We also tested a different competitor: *L. plantarum*, a commonly used probiotic species.

**Figure 5:**
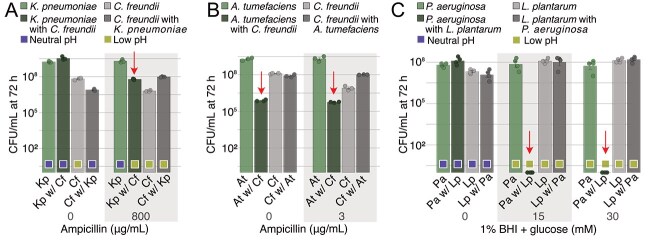
Experiments test the approach with other species pairs. Bars show the mean $\pm $ standard deviation with individual CFU/ml datapoints from the end of 72 h batch cultures (full time-series in Fig. S14). This time-point is a proxy for what would be transfer 1 and may be indicative of the longer-term outcome (Fig. 3). The red arrows indicate cases where the pathogen is suppressed because of the presence of an acidifying competitor species. pH measurement (where measured) is shown in purple and yellow boxes, with the two co-culture bars in the same experiment showing identical color (e.g. pH of Kp w/ Cf is the same as Cf w/ Kp). (A) *C. freundii* (selected for increased resistance to ampicillin) can inhibit the growth of *K. pneumoniae* in co-culture at 800 $\mu $g/ml of ampicillin. Without ampicillin, it is *C. freundii* that grows worse. (B) *C. freundii* (ampicillin-sensitive WT) can inhibit *A. tumefaciens* regardless of the presence of ampicillin. (C) *P. aeruginosa* is inhibited by *L. plantarum* but only if glucose is added to BHI medium. Even without antibiotics, *P. aeruginosa* does not survive until the end of what would be the first transfer (as opposed to with *C. freundii* in Fig. 1C and 3).

*Klebsiella pneumoniae* had a negative effect on the growth of *C. freundii* in the absence of ampicillin. But when 800 $\mu $g/ml of ampicillin were added, *K. pneumoniae* grew significantly worse in co-culture compared with mono-culture (Fig. 5A red arrow). Strong acidification was observed in the mono-cultures of *C. freundii* and in the co-culture with ampicillin. This suggests that *K. pneumoniae* would be eliminated more easily in transfers with ampicillin and *C. freundii* than with ampicillin alone due to the acidification of the medium. Ampicillin would nevertheless be needed in this case.

*Agrobacterium tumefaciens* was also inhibited by *C. freundii* compared with when it was grown alone. In this case, the effect was independent of the presence of ampicillin. Because this experiment was one of the first ones we conducted, the original wildtype *C. freundii* was used, which had an MIC of 3 $\mu $g/ml, and we did not measure the pH. Nevertheless, this experiment suggested that *A. tumefaciens* would be eliminated in a co-culture with *C. freundii*, which we confirmed in transfer experiments (Fig. S15), where *A. tumefaciens* was eliminated faster in the absence of ampicillin (in transfer 2 versus transfer 4 at 3 $\mu $g/ml of ampicillin). The model had suggested that using antibiotics with a sensitive competitor might be detrimental to eliminating the pathogen (Fig. 4C), which might be what we observe with *C. freundii* and 3 $\mu $g/ml of ampicillin.

Having established that different pathogens can be inhibited, we next changed the competitor to *L. plantarum*, a common probiotic species that is known to acidify by producing lactic acid [27]. We did not manage to increase the resistance of *L. plantarum* to antibiotics, nor to grow it in the minimal medium used above. Given our model predictions, though, we anticipated that it would be worth testing its effect on the pathogens even without antibiotics in rich medium with increasing glucose concentrations. As of 15 mM of glucose, *L. plantarum* inhibited *P. aeruginosa* completely, such that no cells were detected after 24 h of a batch co-culture (Fig. 5C). This effect is striking, given that alone, *P. aeruginosa* could grow to an approximate population size of $10^{8}$ CFU/ml. In co-culture with *K. pneumoniae* the effects were more variable (Fig. S16): the first time we ran the experiment, *K. pneumoniae* dropped below the detection limit by 48 or 72 h, at glucose concentrations 30 mM or higher. But when we repeated the experiments two more times, *K. pneumoniae* was not or barely affected by *L. plantarum*. This suggests that antibiotics may be needed in addition to the acidifying species to more robustly suppress *K. pneumoniae* (as in Fig. 5A).

Overall, our model and experiments demonstrate that although different species will have different properties such that the precise conditions in which our approach works will differ, it is in principle not restricted to *C. freundii* and *P. aeruginosa*. We do expect though that in some cases, e.g. if the pathogen is highly competitive or the competitor too sensitive to low pH, it will not be possible to eliminate the pathogen.

## Discussion

How the chemical environment shapes interactions between bacterial species is critical not only to understand microbial community dynamics and function generally, but also to control a species of interest. In previous work, we had shown how to switch the interactions between two bacterial species from negative to positive by changing the chemical environment [18], whereas in this study we apply the control of interactions to eliminate a pathogen through the increase of glucose concentration together with—or instead of—antibiotic administration. This works by feeding the acidifier, which we are calling “feeding the enemy’s enemy,” to reduce the pH thereby inhibiting pathogens that are less sensitive to low pH. By testing this principle on several competitor-pathogen pairs, we demonstrate the generality of the idea.

Our observations build on and connect to the idea that acidification promotes pathogen colonization resistance, which is important in the context of the host microbiome [9] and in food preservation [17]. The idea that a drop in pH caused by the metabolism of certain species can shape species composition and that pH changes can be mediated by resource concentrations has been demonstrated in the lab [14, 19]. Yet few studies have linked this idea to eliminating pathogens [28] and we were lacking a general test of this approach.

Our work also relates to studies that have focused on how interactions between species, such as cross-feeding [8, 29] or cross-protection [6, 30], can reduce the efficacy of antibiotic treatment [5, 31, 32], except that in this case, we are exploiting negative interactions between species to increase its efficacy. We also know that antibiotics and low pH (abiotically) can have synergistic effects in several bacterial species and contexts [20–22, 33–35], because pH is often modified by bacteria and is of great importance to homeostasis [13, 14] and can change the conformation of transport proteins of bacterial membranes [15, 16]. In an applied medical context, the ultimate goal is to reduce the concentrations of antibiotics used to eliminate pathogens. We consider our approach to be highly relevant to counter the rise of antibiotic resistance. In practice, on infection, we are proposing to administer an acidifying probiotic together with additional resources (prebiotics, e.g. glucose) that would increase acidification. However, this approach remains at a proof-of-concept stage and more work is needed to identify its risks and when it would be preferred over current standards of care. As a first point, *C. freundii* would not be a suitable probiotic candidate, given that it can be an opportunistic pathogen itself [36]. We used it here to illustrate the concept. Ideally, we would have used a probiotic species like *L. plantarum* that is clearly nonpathogenic throughout this study, but *L. plantarum* is more challenging to grow in controlled conditions. As an alternative to administering a probiotic species, one could evaluate the presence of preexisting pH-modulating species in the host and stimulate their growth by administering prebiotics. This approach would be particularly interesting for combating plant pathogens like *A. tumefaciens* (Fig. 5B and S15) where the use of antibiotics is restricted in many countries.

Applying “feed the enemy’s enemy” in the context of a host is still a few steps away, though. In the host, pH changes might be tightly buffered and controlled [37], meaning that a competitor species may never be able to acidify the environment significantly. Another complexity of a real environment is that other microbial species are likely to also be present and may be affected positively or negatively by the changes in pH and in turn also affect the targeted pathogen. Nevertheless, in the spatially structured environment of the host tissue, it has been suggested that acidification can act locally to inhibit pathogens [10].

Two additional factors can be important in shaping interactions and would be interesting to explore in future work: the role of spatial organization, and the evolution of resistance (Fig. S10). Regarding spatial structure, the extinction of *P. aeruginosa* depended on frequently mixing and physically disturbing the cultures, whereas leaving them undisturbed almost eliminated the antibiotic effect (Fig. S4  S5). Our current hypothesis is that *P. aeruginosa* may have formed biofilms in the undisturbed cultures as a response to antibiotic stress [23], which protected them against the antibiotic [24, 25]. *P. aeruginosa* also forms more biofilm and gains resistant faster in the acidic conditions of cystic fibrosis lungs [26], which corroborates our observations.

Our findings highlight the importance of considering the community and chemical context of pathogens during antibiotic treatment of microbial infections. In most cases, the outcome of antibiotic treatment is driven by several dynamic variables that are the direct result of bacterial interactions and growth, including the pH. We demonstrate that the strength of these interactions, such as the degree of acidification, can be externally controlled by manipulating the chemical environment, in this case the addition of glucose. A deeper understanding of such variables is key to controlling microbial interactions, thereby providing an alternative strategy to enhance or in some cases even replace the use of antibiotics. Our work advocates for studying the effect of antimicrobial compounds in environmental conditions that better mirror the complexity of bacterial communities [38], particularly in this broader framework of community control so that we can increase our ability to obtain successful treatment outcomes.

## Supplementary Material

Supplementary_material_wrag170

## Data Availability

All experimental data and code are available on Zenodo (DOI: 10.5281/zenodo.17751754).
