# Supplementary material for: Acidifying bacteria inhibit pathogenic bacteria more strongly with increasing glucose
Source: ISME J. 2026 Jun 27;20(1):wrag170. doi: 10.1093/ismejo/wrag170 (PMC13477223; doi:10.1093/ismejo/wrag170)
Supplement: Supplementary file 1 [file supplementary_material_wrag170.zip › dosSantos_supplement_wrag170.pdf]

Supporting information for “Acidifying bacteria inhibit pathogenic bacteria more strongly with increasing glucose”

Dos Santos, Vulin & Mitri

S1 Mathematical model

To better understand the dynamics that we observed within our 2-species community (Fig. 1), we built a consumer-resource model based on modified Monod equations (with maximum growth rate r, half-saturation constant K, and yield Y). In our model, species B_1 (pathogen) and B_2 (competitor) can grow together using the resource C and produce a certain quantity of organic acid A that is proportional to the quantity of C consumed. Species B_1 produces a very small quantity of A whereas B_2 produces a significant quantity of A (described by the parameter a_i) that will acidify the medium through the net production of protons H (directly used to calculate the pH using equation S6). Species B_1 can consume a fraction q of organic acid A through cross-feeding. The value of H is obtained after applying a neutralisation function (equation S7) that mimics the effect of a chemical buffer (used in our growth medium) on the concentration of organic acid A. Species B_1 and B_2 have a sensitivity to acidic pH that is described by equation S8 [1] that depends on 2 parameters: pK_h and pK_l which are respectively the high and low pH bounds, representing the pH value above and below which the species will start being sensitive to the pH, respectively. By default, species B_1 is sensitive to pH below 5.5 and B_2 below 3.5. In addition, there is a concentration b of antibiotic that affects B_1 and B_2 and depends on the sensitivity parameter l_i, which induces a lag in bacterial growth. B_1 has a high sensitivity to ampicillin whereas B_2 is mostly resistant.

System of ordinary differential equations (ODEs)

$$\frac{dB_1}{dt} = r_{1,1}\frac{C}{C+k_{1,1}}\frac{t^2}{(l_1 b)^2+t^2}F_1(pH(H))B_1 + r_{1,2}\frac{qA}{qA+k_{1,2}}\frac{t^2}{(l_1 b)^2+t^2}F_1(pH(H))B_1 \quad \text{(S1)}$$

$$\frac{dB_2}{dt} = r_{2,1}\frac{C}{C+k_{2,1}}\frac{t^2}{(l_2 b)^2+t^2}F_2(pH(H))B_2 \quad \text{(S2)}$$

$$\frac{dC}{dt} = -\frac{1}{Y_{1,1}}\frac{r_1}{C+k_1}\frac{t^2}{(l_1 b)^2+t^2}F_1(pH(H))CB_1 - \frac{1}{Y_{2,1}}\frac{r_2}{C+k_2}\frac{t^2}{(l_2 b)^2+t^2}F_2(pH(H))CB_2 \quad \text{(S3)}$$

$$\frac{dA}{dt} = (\sum a_i \frac{1}{Y_i}\frac{r_i}{C+k_i}\frac{t^2}{(l_i b)^2+t^2}F_i(pH(H))CB_i) - \frac{1}{Y_{1,2}}\frac{r_{1,2}}{qA+k_{1,2}}\frac{t^2}{(l_1 b)^2+t^2}F_1(pH(H))qAB_1 \quad \text{(S4)}$$

$$\frac{dH}{dt} = N(pH(H))(1-q)\frac{dA}{dt} \quad \text{(S5)}$$

Where the pH is:

$$pH(H) = \frac{1}{2}(pKa - log(H)), \quad \text{(S6)}$$

which is the general formula to calculate the pH of a weak acid. Here the pKa always takes the value 9.

The neutralisation (buffering) function is:

Default species-specific parameters		B_1 $(i = 1)$		B_2 $(i = 2)$
**Parameter**	**Description**	Glucose $(j = 1)$	Organic acid $(j = 2)$	Glucose $(j = 1)$
$r_{i,j}$	growth rate	0.45	0.45	0.4
$k_{i,j}$	half saturation constant	8	5	8
$Y_{i,j}$	yield	10000	40000	10000
l_i	lag factor	15		0.01
a_i	acidification rate	10^{-15}		0.2
pK_{li}	lower pH bound	5.5		3.5
pK_{hi}	higher pH bound	8.5		8.5

Table S1: List of all species specific parameters and their values

Default global parameters		
**Parameter**	**Description**	**Value**
b	antibiotic concentration	0.3
q	fraction of cross-fed organic acid	0.1
t	time	from simulation
pKa	acid dissociation equilibrium	9
m	slope of neutralization	-5
x_0	midpoint of neutralization	5.5

Table S2: List of all global parameters and their values.

$$N(pH(H)) = \frac{1}{1 + e^{-m(pH(H)-x_0)}} \tag{S7}$$

This logistic function depends on the pH and takes a value between 0 and 1 depending on the pH (0 = maximum buffering capacity, 1 = no buffering capacity). The change in organic acid A is multiplied by this term, which cancels a fraction of A, thereby buffering the medium. Here, m always takes the value -5 and x_0 always takes the value 5.5.

The pH inhibition function is defined as (from [1]):

$$F_i(pH(H)) = \frac{1 + 2 * 10^{0.5(pK_{li}-pK_{hi})}}{1 + 10^{(pH-pK_{hi})} + 10^{(pK_{li}-pH)}} \tag{S8}$$

This bell-shaped function depends on the pH and can take values between 0 and 1 (0 = full inhibition, 1 = no inhibtion). The growth of B_1 or B_2 is multiplied by this function, which cancels a fraction of growth.

The model was implemented in Julia 1.8.5. ODEs were solved with the package `DifferentialEquations` using the method `Rosenbrock23`, which is an order 2/3 L-Stable Rosenbrock-W method, appropriate for very stiff equations with oscillations at low tolerances.

References

[1] Angelidaki, I., Ellegaard, L., Ahring, B. K. (1993). *A mathematical model for dynamic simulation of anaerobic digestion of complex substrates: Focusing on ammonia inhibition.* Biotechnol. Bioeng. 42, 159–166.

Supplementary figures

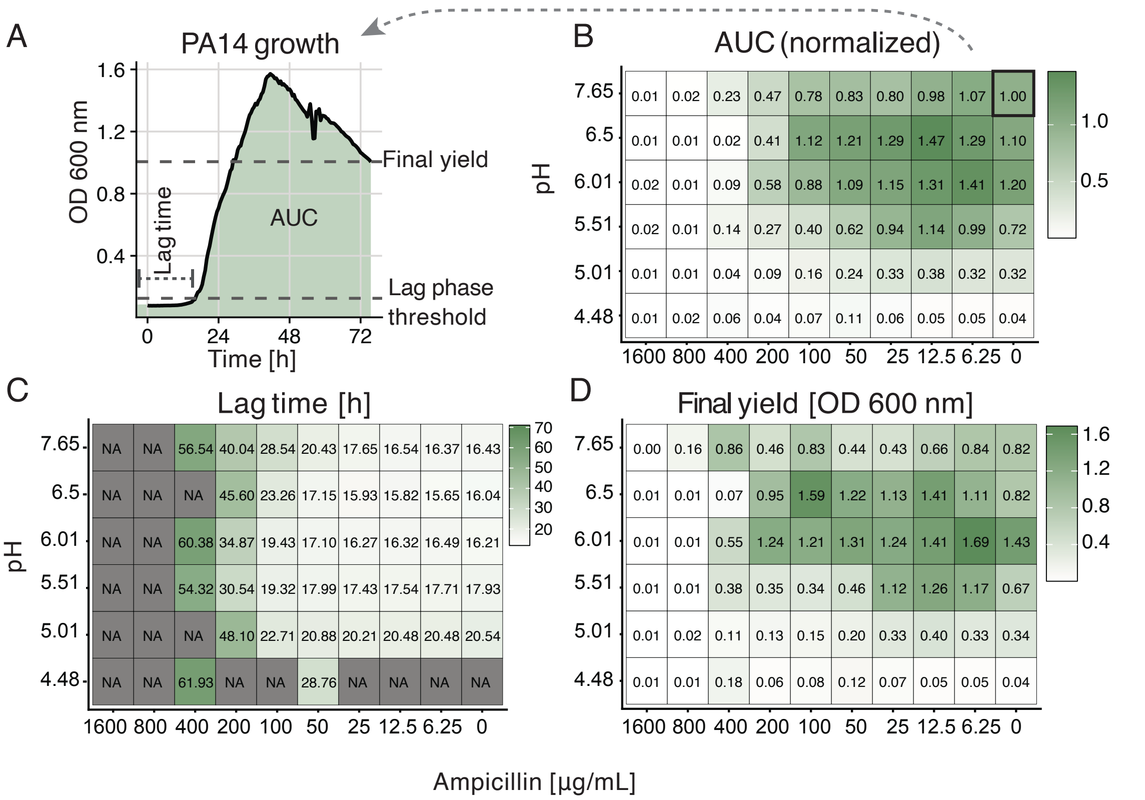

Figure S1: Effect of ampicillin combined to acidification on *P. aeruginosa*. (A) Growth of *P. aeruginosa* in non-acidified medium without ampicillin. The different growth parameters considered in the following panels are displayed. An ampicillin- combined with pH-sensitivity assay was performed and the normalized area under the growth curve (AUC) (B), the lag time (C), and the final yield (D) were determined. “NA” in panel (C) indicates “not applicable”, because the OD_{600} never exceeded the threshold, here: 0.09.

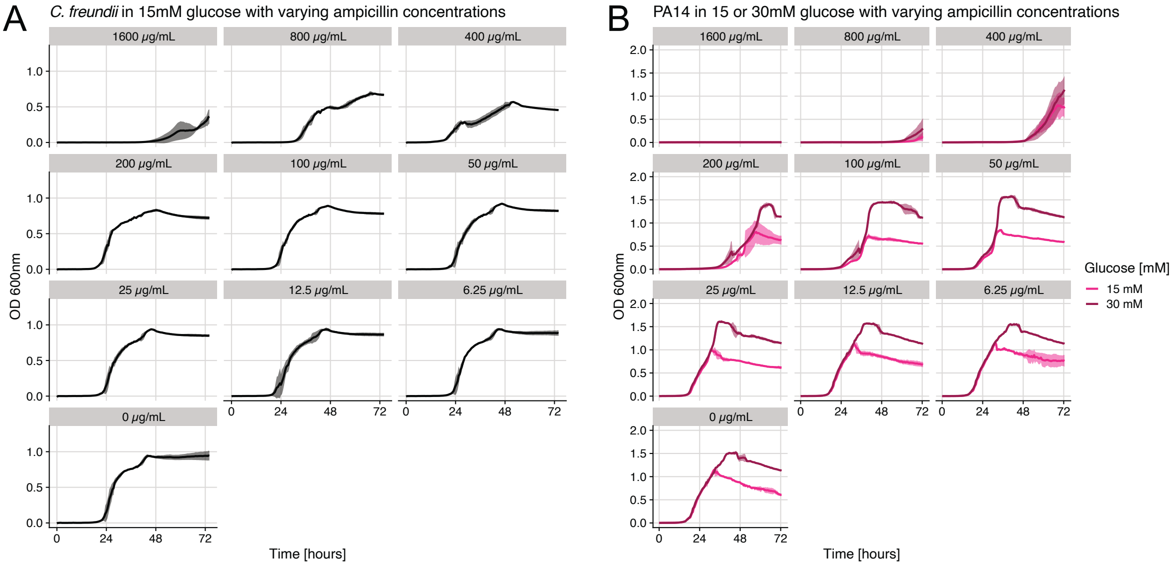

Figure S2: (A) Growth of *C. freundii* (isolate selected for resistance against ampicillin, not WT) in varying concentrations of ampicillin in minimal medium with 15 mM of glucose. (B) MIC of *P. aeruginosa* in varying concentrations of ampicillin in minimal medium with either 15 or 30 mM of glucose. For all curves, OD_{600} was measured every 30 min for approximately 72 hours (n = 3).

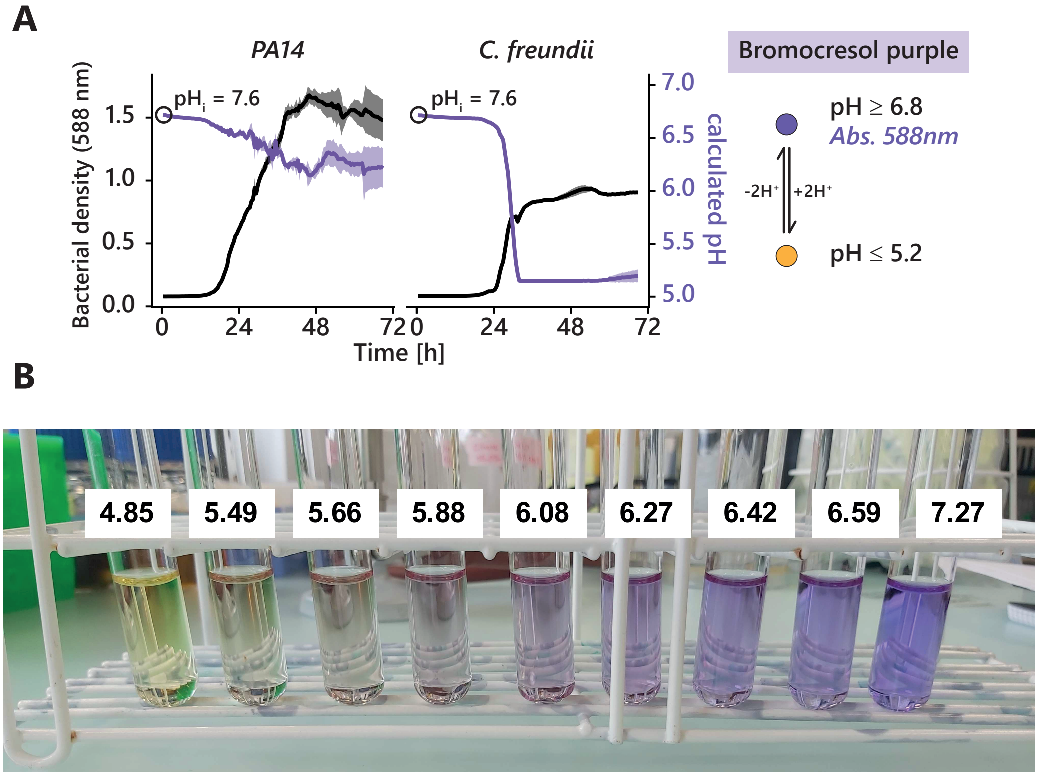

Figure S3: Use of the pH dye bromocresol purple to determine the pH of bacterial cultures. (A) Growth curve and pH curves of *P. aeruginosa* and *C. freundii* grown in minimal medium with 30 mM of glucose. The medium contained 0.001 % (w/w) of bromocresol purple (BP). Bacterial density was measured at 750 nm and the absorbance of BP was measured at 588 nm and corrected to remove bacterial density, (n = 3). For more details see methods. (B) Color range of BP in cell-free liquid with pH adjusted as indicated.

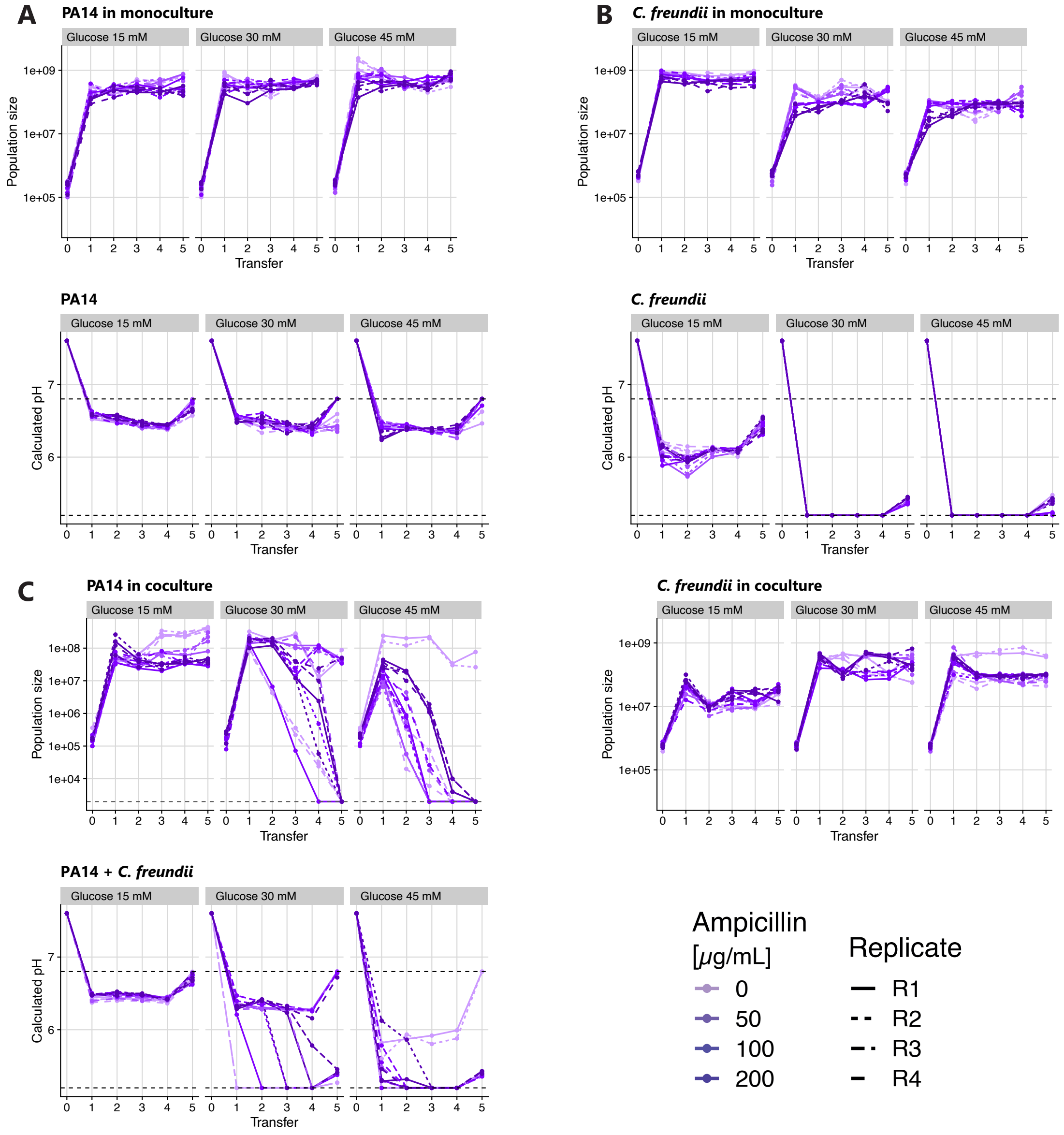

Figure S4: Approximately equal initial population size of *P. aeruginosa* and *C. freundii* (both initial OD_{600} of 0.001), antibiotic gradient up to 200 μg/mL. Growth of *P. aeruginosa* (A) and *C. freundii* (B) over 5 transfers (3 days of growth, 1 % transfer in fresh medium, CFUs at each transfer) in mono-culture (top row) and pH value at each timepoint from the absorbance of bromocresol purple measured at OD_{588} at each transfer (bottom row). (C) Growth of *C. freundii* and *P. aeruginosa* when they are co-cultured as well as the pH values (same as in Fig. 3C).

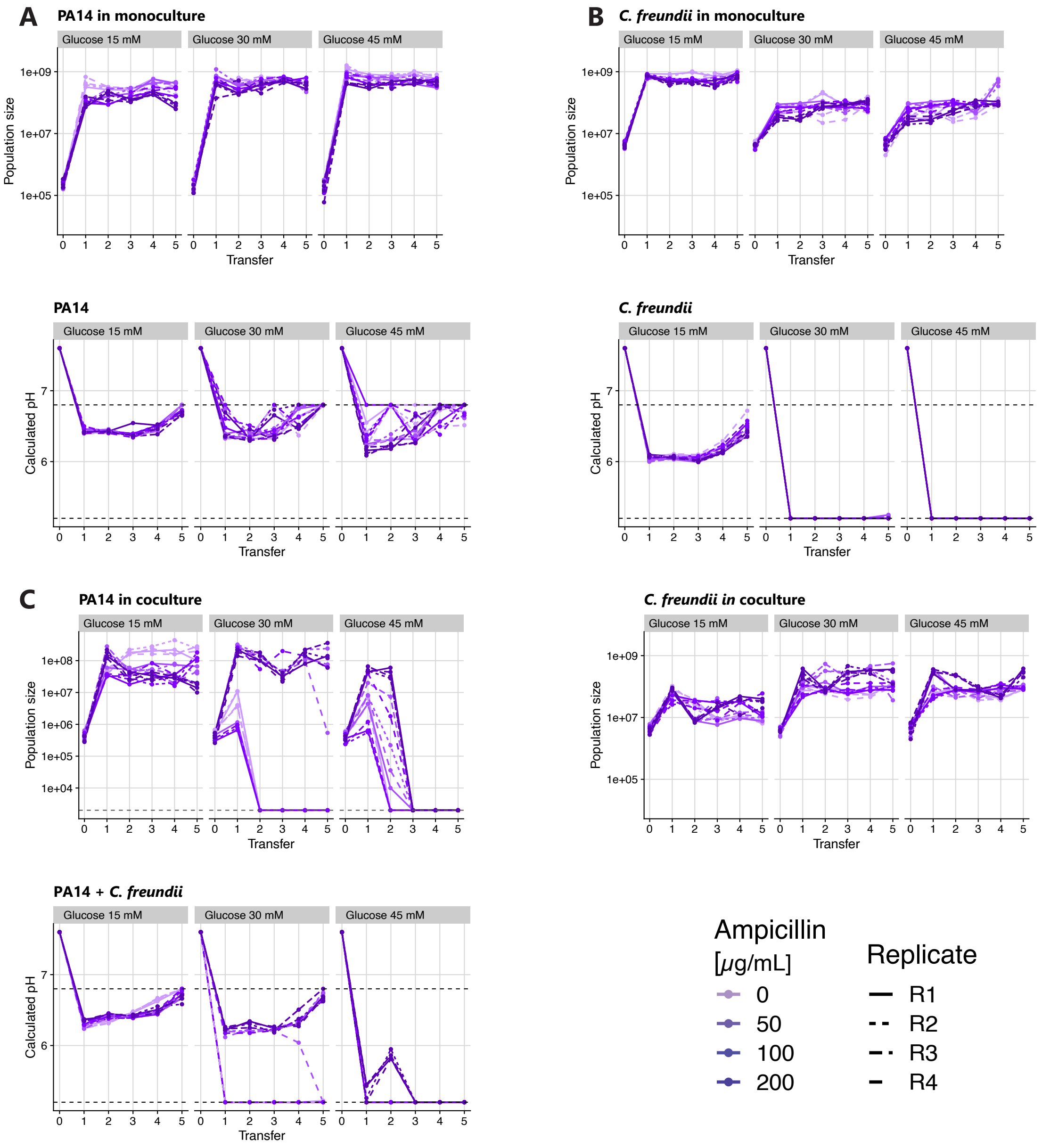

Figure S5: Same as the experiment shown in Fig. S4 but the initial OD_{600} of *C. freundii* (0.01) is 10× higher than *P. aeruginosa* (0.001), antibiotic gradient up to 200 μg/mL. Growth of *P. aeruginosa* (A) and *C. freundii* (B) over 5 transfers (3 days of growth, 1 % transfer in fresh medium, CFUs at each transfer) in monoculture (upper graph) and pH value at each timepoint from the absorbance of bromocresol purple measured at OD_{588} at each transfer (lower graph). (C) Growth of *C. freundii* and *P. aeruginosa* when they are cocultured as well as the pH values (same as in Fig. 3C).

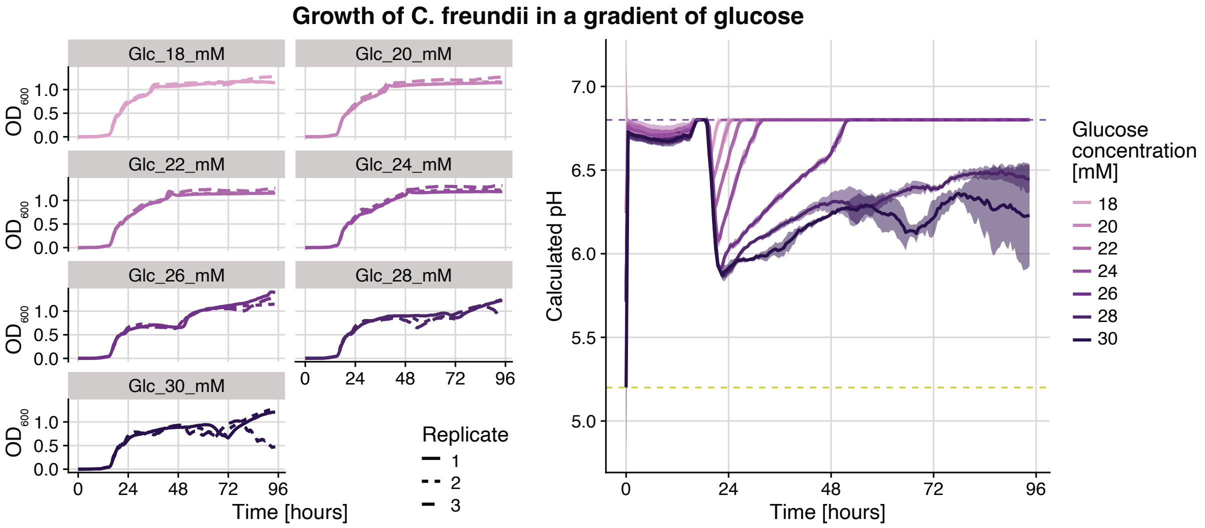

Figure S6: Growth and acidification by *C. freundii* in a gradient of glucose to determine whether there was a concentration at which acidification patterns over 72 h were substantially different from 15 mM and from 30 mM. On the right, the drop and then recovery of the pH back to 6.7 is due to the effect of the buffer (M9) in our minimal medium. This helped us to choose the concentration of 26 mM for our repeated transfer experiment, where the buffer still recovers a neutral pH but the pH drops and stays low for around 24h, which we estimated would have a significant effect on *P. aeruginosa*.

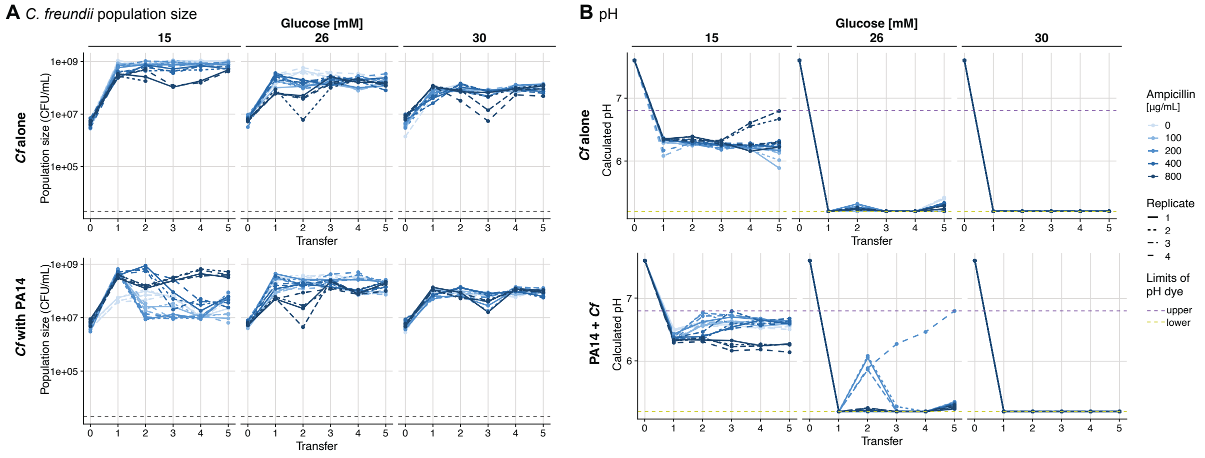

Figure S7: Data for *C. freundii* from the experiment corresponding to the data shown in Fig. 3. These data show that *C. freundii* acidifies the medium more with increasing glucose concentration when alone, which has a negative effect on its population size (it suffers from its own acidification). It grows similarly at all antibiotic concentrations. In co-culture with *P. aeruginosa*, it grow less well in cases where *P. aeruginosa* grows a lot (at low glucose and low antibiotic concentration) and when it grows less, the pH also drops less.

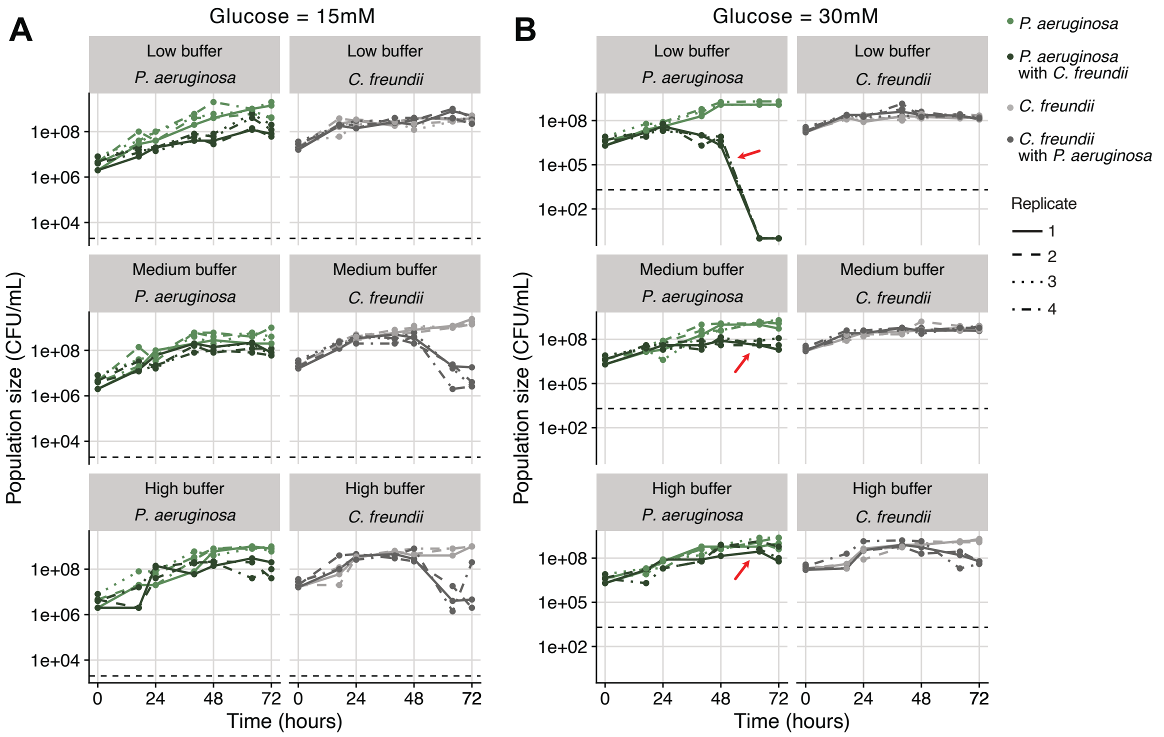

Figure S8: The effect of buffering capacity on the interaction between *P. aeruginosa* and *C. freundii*. For low buffer (top row), 30 g/L Na_2HPO_4 and 15 g/L KH_2PO_4 were used to prepare the M9, for medium buffer (middle row) 60 g/L Na_2HPO_4 and 30 g/L KH_2PO_4 were used, whereas for high buffer (bottom row), M9 was prepared using 120 g/L Na_2HPO_4 and 60 g/L KH_2PO_4. Here, we ran 72 h batch cultures at 15 mM or 30 mM glucose using mono- or co-cultures of *P. aeruginosa* and *C. freundii*. The red arrows show the effect we were aiming to test: that lowering or increasing the buffering capacity of the medium (compared to the baseline "medium buffer" used throughout the paper) can greatly affect the population size of *P. aeruginosa* when in co-culture with *C. freundii*, supporting our main argument that pH is the main mediator of their interaction.

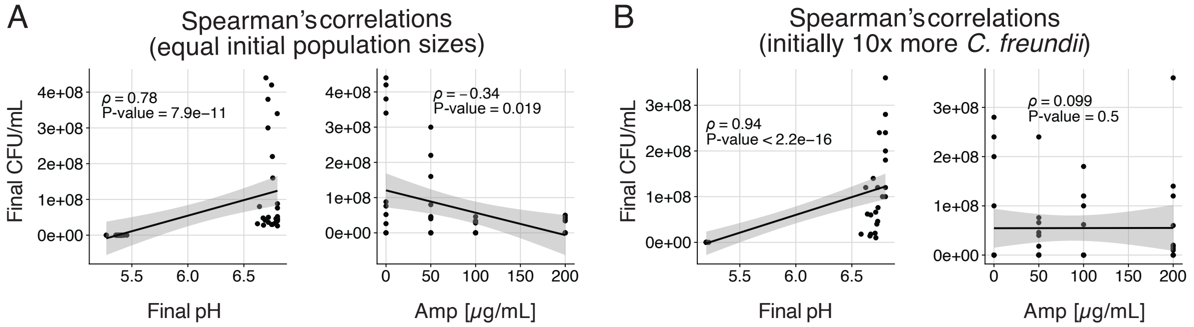

Figure S9: **(A)** From the data in Fig. S4, Spearman's rank correlation between the final population size and the final pH (transfer 5) (left) and the ampicillin concentration (right). **(B)** From the data in Fig. S5, Spearman's rank correlation between the final population size and the final pH (transfer 5) (left) and the ampicillin concentration (right). We used the command `cor.test(..., method = "spearman")` in R to obtain ρ and P values.

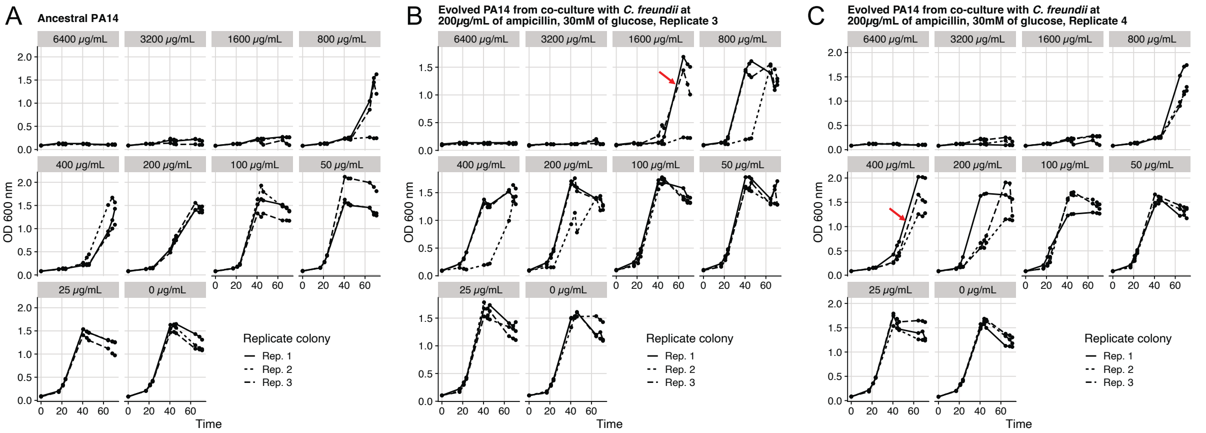

Figure S10: MIC against ampicillin of (A) the ancestral strain of *P. aeruginosa* compared to some of the isolates from the fifth transfer in two experimental replicates (B) and (C) where they had survived in co-cultured with *C. freundii* at 30 mM of glucose and 200 μg/mL of ampicillin. We show three individual colonies that were isolated and grown separately in minimal medium with 30 mM of glucose and the OD_{600} was measured 3 times per day for 3 days in a microplate reader (n = 3). Whereas the ancestral *P. aeruginosa* has an MIC of 800 μg/mL, some of the isolates from the end of the transfers, but not all, showed a higher MIC of up to 1600 μg/mL. Increased resistance to ampicillin is highlighted in the curves indicated with red arrows. Resistance was not universal, though, showing that survival could also be due to tolerance.

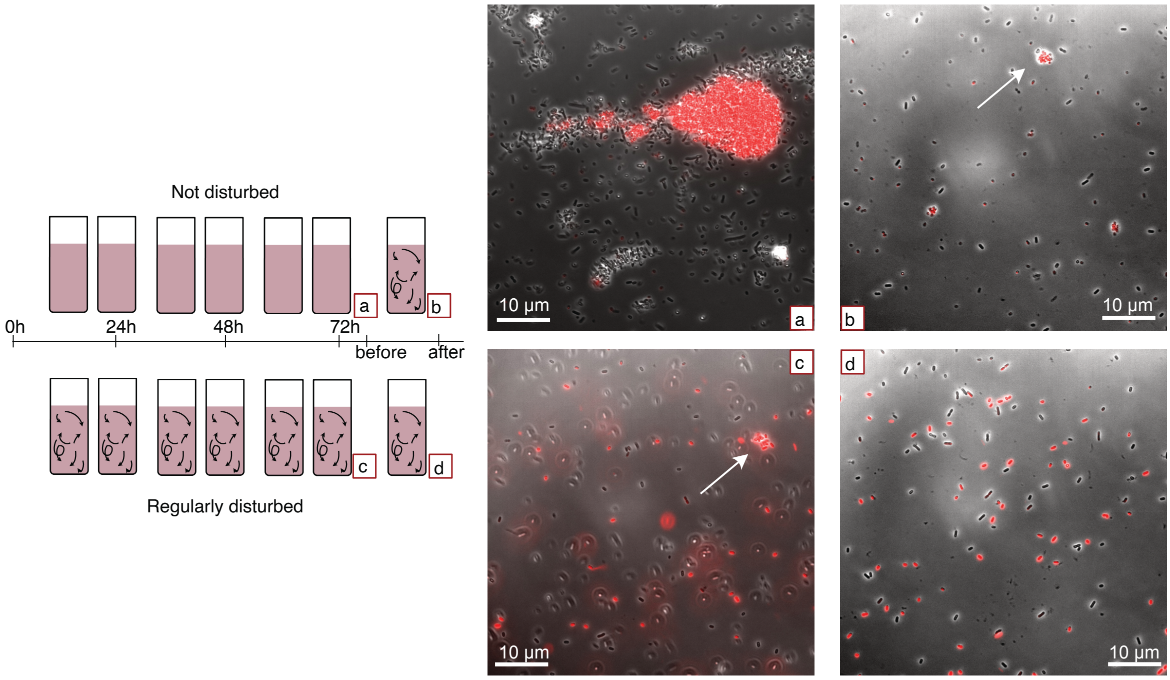

Figure S11: Observed biofilm formation in *P. aeruginosa*. In a co-culture of *C. freundii* and *P. aeruginosa* (30 mM glucose), cells showed minimal clumping when not regularly disturbed. Cells were grown for 72 h after inoculation, either without disturbance, or disturbed twice a day by pipetting. After this incubation period, samples were obtained and imaged by microscopy before or after an ultimate disturbance (right). We observe larger clumps in undisturbed conditions (a) than in disturbed conditions (c). These clumps were small (white arrows) and dispersed upon disturbance. *P. aeruginosa* is fluorescently labelled with mCherry, scale bar: 10 µm. This was not quantified systematically.

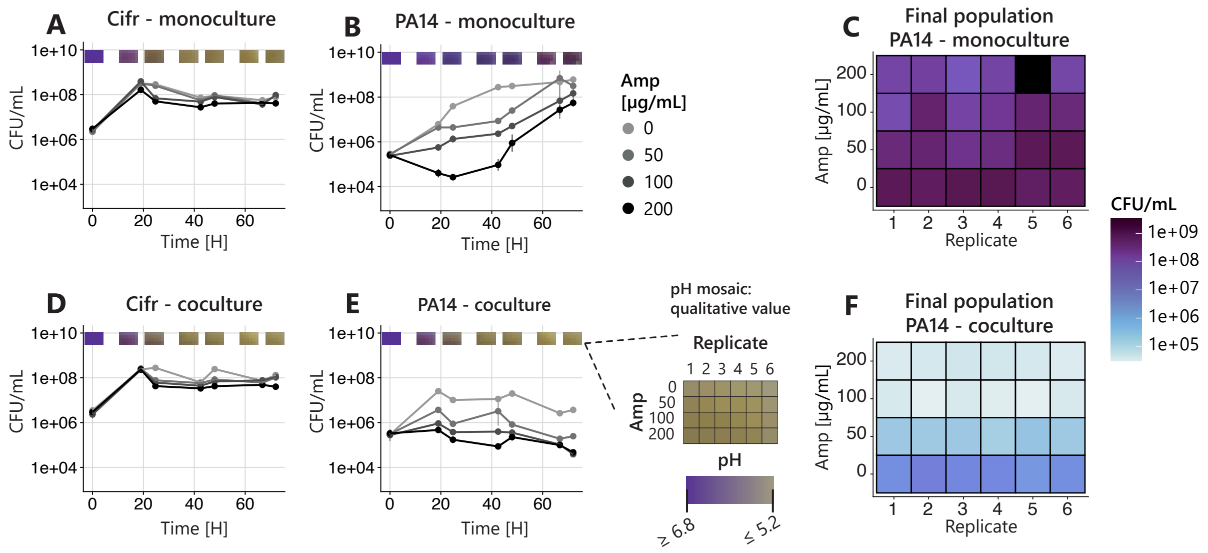

Figure S12: Experiment to test the effect of frequent disturbances to the culture. *P. aeruginosa* and *C. freundii* were grown in mono- and coculture (30 mM glucose), but rather than leaving them undisturbed for 72 h, CFUs were sampled twice per day (except on the day of inoculation) for 72 h. pH was visually estimated using bromocresol purple and taking pictures at each sampling time. For clearer data, we performed a binary thresholding where we assigned one of 2 colors to each sample: purple for no acidification and yellow for acidification. Any culture that was in the process of turning yellow (moment of acidification) and displayed a brown/yellow color was considered as acidified, otherwise it was considered as not acidified. **(A)** Monocultures of *C. freundii.* **(B)** Monocultures of *P. aeruginosa.* **(C)** Final population of *P. aeruginosa* in monoculture from panel B. **(D)** *C. freundii* when in coculture with *P. aeruginosa.* **(E)** *P. aeruginosa* when in coculture with *C. freundii.* **(F)** Final population of *P. aeruginosa* from panel E. This experiment showed us that ampicillin had the expected inhibitory effect on *P. aeruginosa* if the cultures were disturbed and motivated us to repeat the transfer experiment again with regular disturbances.

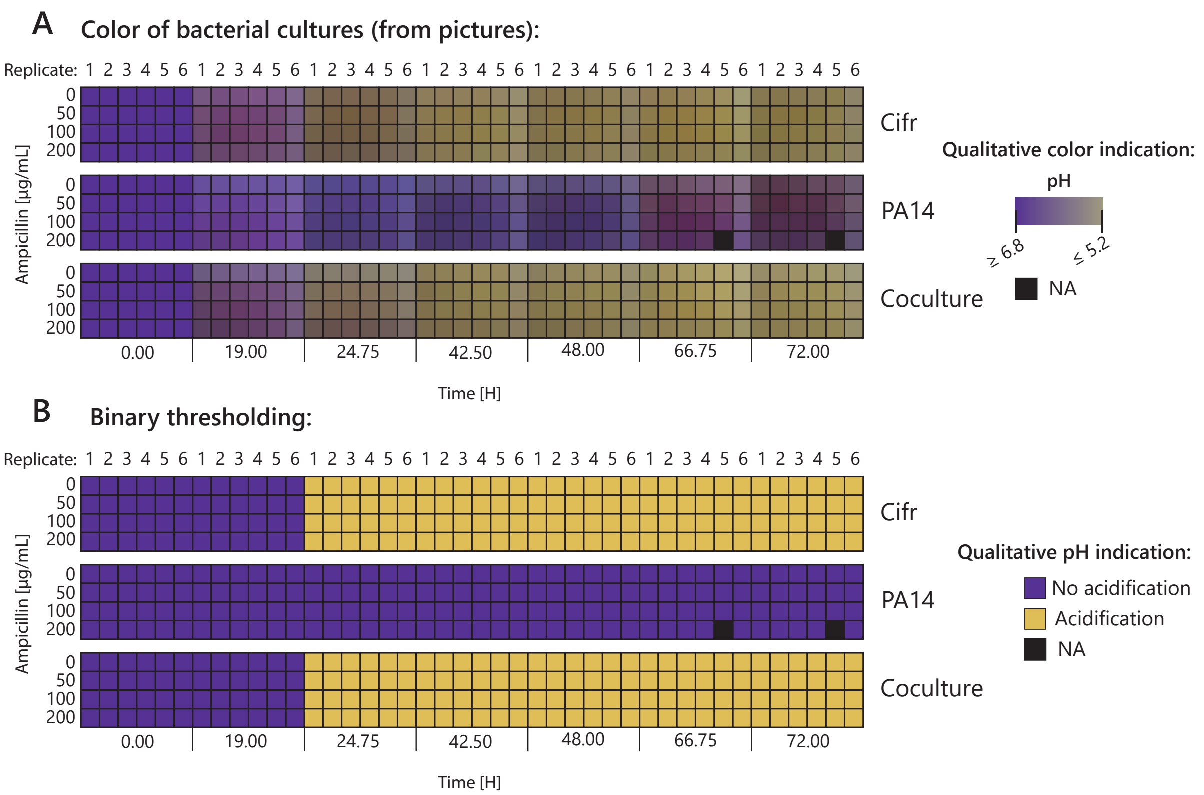

Figure S13: Acidification data from Fig. S12 (A) Colors of the bacterial cultures containing bromocresol purple obtained from pictures taken before each CFU sampling. The colors of the bacteria affect the color of the culture (white/brown for *C. freundii* and pink for *P. aeruginosa* because it contains an m-Cherry tag). (B) For clearer data, we performed a binary thresholding where we manually assigned only 2 colors to each sample based on panel A: purple for no acidification and yellow for acidification. Any culture that was in the process of turning yellow (moment of acidification) and displayed a brown/yellow color was considered as acidified, otherwise it was considered as not acidified.

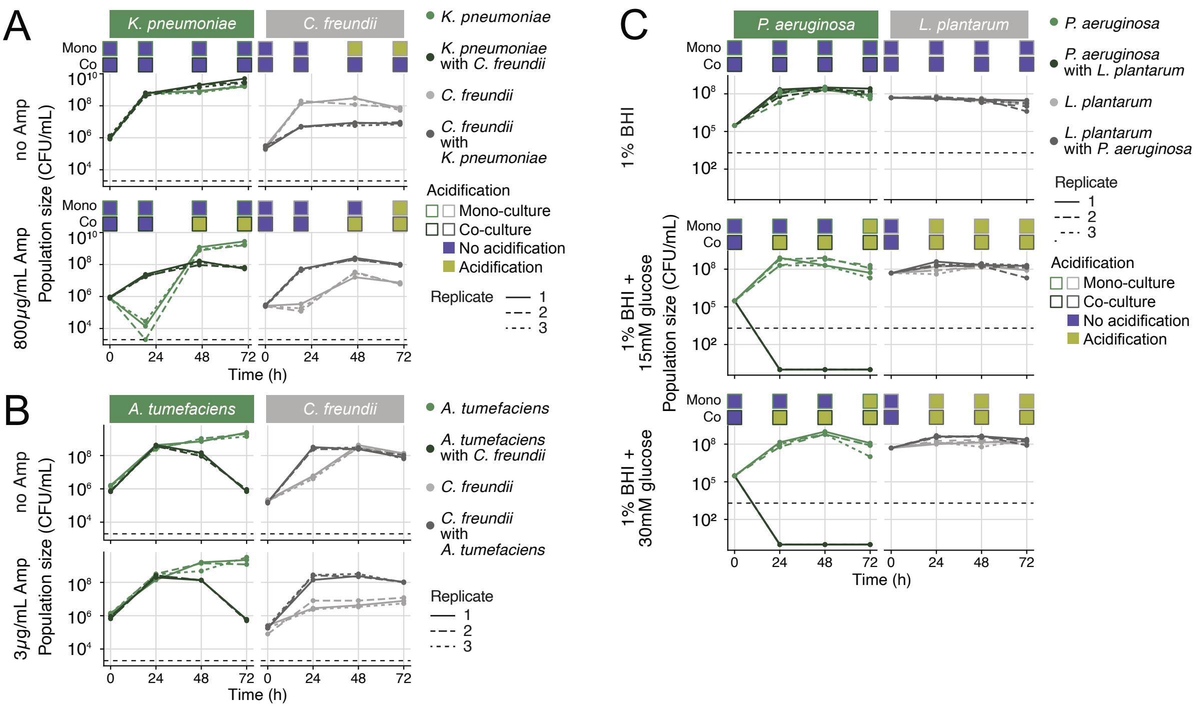

Figure S14: Same data as Fig. 5 but where all sampled time-points are shown in the 72 h batch culture. Fig. 5 instead only shows the 72 h time-point.

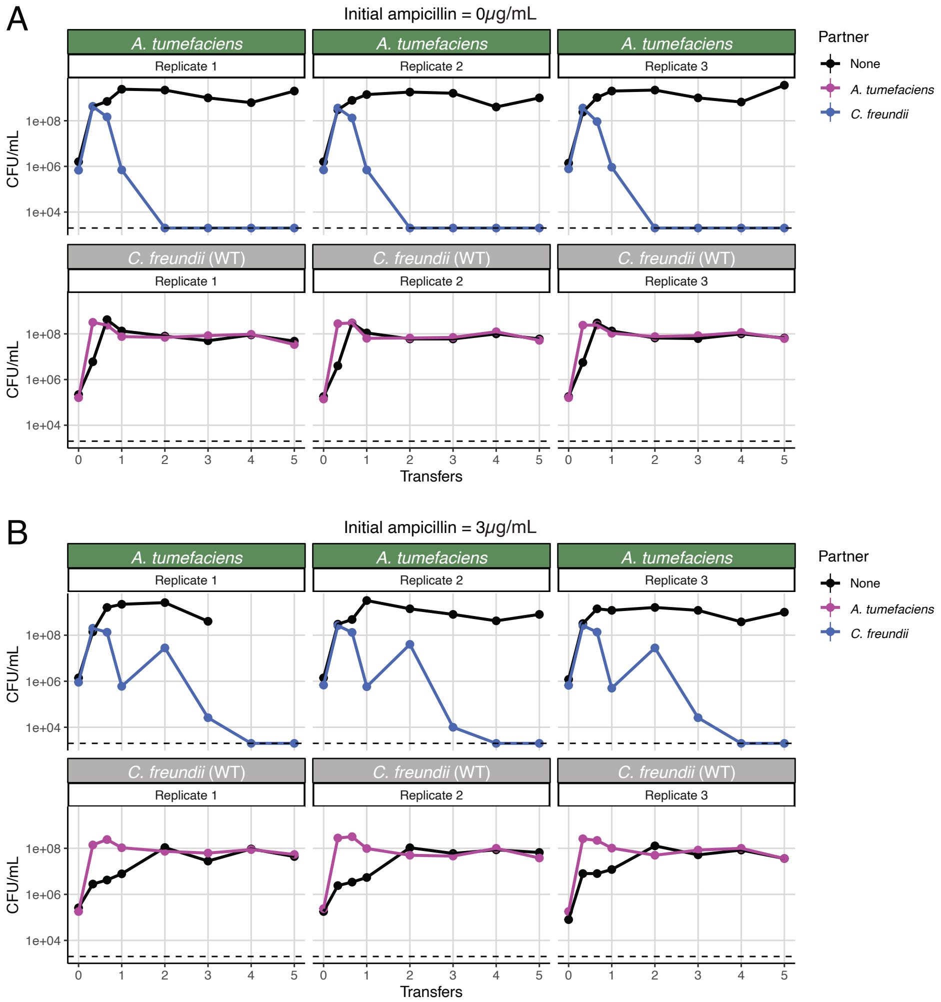

Figure S15: Growth-and-dilution cycles for *C. freundii* and *Agrobacterium tumefaciens* as the pathogen. We performed 5 transfers at a 100× dilution every 72 h, as in the experiment with *P. aeruginosa* (no additional disturbance). We tested two concentrations of ampicillin: 0 and 3 μg/mL in panels (A) and (B) respectively. *A. tumefaciens* was eliminated by *C. freundii* at both ampicillin concentrations. It even took longer at 3 μg/mL, because *C. freundii* is more sensitive to ampicillin. This is because this is the WT *C. freundii*, before we had selected a more resistant isolate, which is the one used with *P. aeruginosa*.

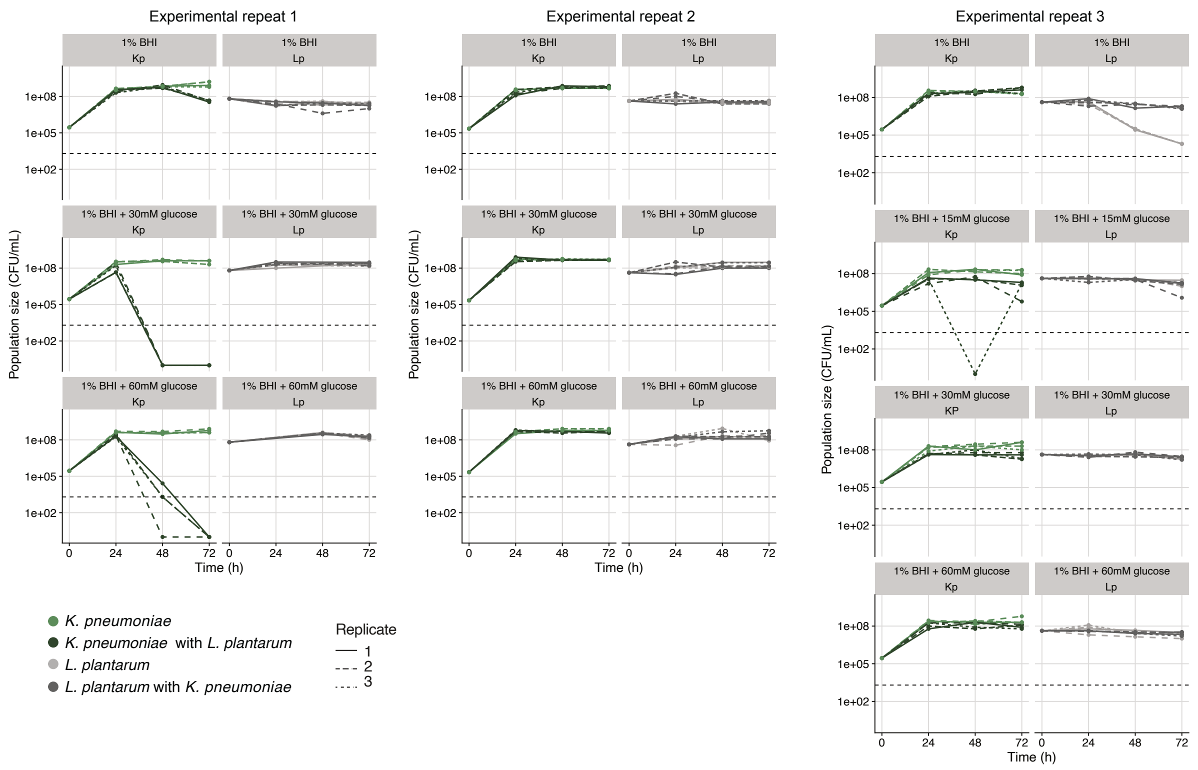

Figure S16: Mono- and co-cultures (light and dark respectively) of *Lactobacillus plantarum* as the competitor and *Klebsiella pneumoniae* as the pathogen (gray and green respectively) in single batch cultures over 72 hours (no transfers). The figure shows three repeats of the experiment with different glucose concentrations shown in the rows. In the first experimental repeat, *K. pneumoniae* was eliminated at 30 and 60 mM of glucose added to BHI, but not when glucose was not added. On repeating the experiment, the effect disappeared. This appears to match our observation of *K. pneumoniae*'s growth with *C. freundii*: antibiotics were needed to have a robust inhibition of the pathogen. In this case, *L. plantarum* is highly sensitive to the antibiotic, which could therefore not be used. Although we measured pH patterns with the dye, we did not add them to the figure because in all three experiments we observed no acidification without the addition of glucose, and acidification in the presence of glucose, regardless of the species composition or the concentration of glucose (as long as it was > 0).
